# Metallic foreign body in middle ear: an unusual cause of hearing loss

**DOI:** 10.1186/1746-160X-3-23

**Published:** 2007-05-16

**Authors:** Anna Eleftheriadou, Thomas Chalastras, Dionysios Kyrmizakis, Sotirios Sfetsos, Konstantinos Dagalakis, Dimitrios Kandiloros

**Affiliations:** 1 Department of Otolaryngology, G. Gennimatas Hospital, Athens Greece; 2 Department of Otolaryngology, University of Athens, Hippokration Hospital, Athens Greece; 3 Department of Otolaryngology, General Hospital of Rethimno, Crete

## Abstract

This is a rare case report of a foreign metallic body found in the middle ear. During the use of an electric welding by a metalworker, a glowing drop of dissolved metal overrun, burning the skin of his external auditory meatus, perforated the tympanic membrane and finally was implanted around the ossicles as a foreign body. Due to difficulty of the physical examination and the moderate symptoms (hearing loss and sense of fullness), the foreign body was detected six months after the incident, by CT scanning and it was removed by a transcanal approach under general anesthesia. A successful ossiculoplasty-tympanoplasty was followed four weeks later.

## Background

Foreign bodies of the external ear canal are relatively common in medicine. They have been usually reported in children. On the contrary, foreign bodies in the middle ear are rare. They usually are found as a result of a violent perforation of the tympanic membrane and transfer of the foreign body through the external auditory canal to the middle ear space. Incidents with welding bead injuries are not common in welders in comparison with eye injuries. Nevertheless, it is recommended to wear earplugs for protection. There are some publications available, which describe such incidents. [1-3]

## Case presentation

A 36-year-old metalworker presented at the emergency department with severe otalgia and bleeding from his right ear. He referred that he had an occupational accident when he was operating an electric welding, above his head; a glowing metallic drop of liquified metal was fallen in his right external meatus.

On inspection, the entrance of the external auditory canal was obstructed by massive edema while severe skin erythema with some blood clots were also revealed. Otomicroscopy of the external auditory meatus and eardrum were unfeasible because of the severe obstruction and sharp pain. The tuning forks tests were compatible with conductive hearing loss on the right side. No sensation of vertigo was reported. The rest of head and neck examination was unremarkable. Lesions were treated as severe burn. That included broad-spectrum systemic antibiotics, local applications of antiseptic solutions and creams, analgesics and corticosteroids orally. Three months later, the external auditory meatus appeared dry, atrophied, with scars resulting to a relative stenosis. The tympanic membrane appeared shrunken and thickened bearing a moderate in size perforation at the pars tensa without any secretion. Patient did not complain of pain any more. He only reported a deterioration of the fullness and hearing loss.

Six months later, during a follow up visit, patient was still complaining of the same symptoms. Audiometric evaluation revealed moderate conductive hearing loss on the right ear.

**Figure 1:**
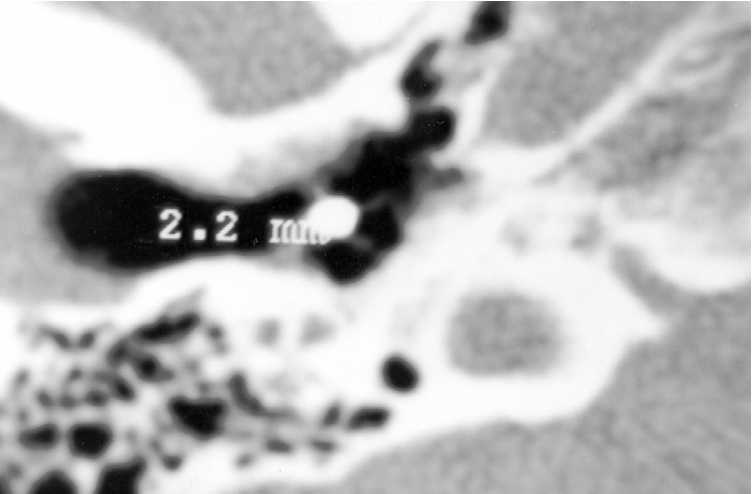
Coronal CT scan demonstrating a metallic foreign body in middle ear.

The patient had undergone a CT scan, where a metallic foreign body was depicted in the middle ear cavity, wedged around the ossicles. The diameter of the foreign body was estimated about 2.2 mm (Figure 1). This small piece of metal was apparently the glowing drop, which had caused the injury few months ago.

**Figure 2:**
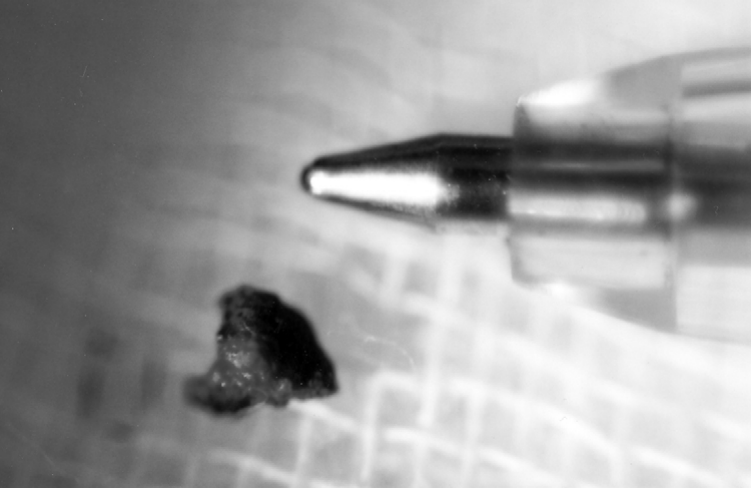
Dimensions of the removed metallic foreign body compared with the nib of a pencil.

An exploration of the middle ear and the ossicular chain under general anesthesia was deemed necessary. Using a transcanal approach, scaring tissue of the external auditory canal was removed using the CO2 laser. A fracture of malleus' manubrium was found as well as adhesions around the remnants of the manubrium, the long process of incus and the head of the stapes. The foreign body was revealed among the adhesions and was removed (Figure 2). An ossiculoplasty- tympanoplasty was followed, four weeks later. Six months after the incident, the perforation was healed and the air-bone gap was disappeared. One year later, the tympanic membrane was still whole and intact and the pure tone audiogram was normal.

## Discussion

In the current literature, very few cases of foreign bodies in middle ear are reported. Syms and Nelson [4] presened 4 cases of impression-material foreign bodies in middle ear induced chronic otitis media. Shimanski [5] reported a case of silicone foreign body in the middle ear caused by auditory canal impression in hearing aid fitting. Patient was complaining of acute otalgia. Kohan *et al*. [6] performed a retrospective analysis of complications after hearing aid fitting and they described six cases of patients suffered from severe complications due to improper ear mold fitting, requiring surgical intervention.

Foreign bodies, particularly from metal, of the middle ear are not often reported but they can induce symptoms like hearing loss, fullness, tinnitus and vertigo. Simons and Eibling [3]described the unique finding of retained slag in the context of a persistent tympanic membrane perforation from a welding injury that occurred more than 30 years ago. Panosian et al [2]reported transtympanic facial nerve injury in welders. Similarly, facial paralysis and hearing loss is presented by Stage and Vinding [7] due to metal spark perforation of the tympanic membrane. In our case the conductive hearing loss was more severe than could be expected in accordance with the perforation's size. CT was very helpful to reveal the cause of the patient's symptoms. In the literature, it is recommended that welding bead injuries tend to heal very badly [8] and recurrent perforations after tympanoplasty are often [1]. In our case, the speedy course of the healing process after the surgical intervention was probably due to the young age of the patient and the relatively early reparation after the injury.

In conclusion a foreign metal body in the middle ear is a rare event but in welders who do not use ear plugs for protection, a penetrated metallic foreign body of the middle ear can cause severe damage. If the anamnesis and the clinical symptoms lead to the suspicion of having a metallic foreign body into the middle ear, we have to recommend X-ray or CT imaging to verify the diagnosis before surgery. Although ocular injuries from welding are more frequent, physicians have to emphasize ear protection, too.
